# Sequence and structural analysis of BTB domain proteins

**DOI:** 10.1186/gb-2005-6-10-r82

**Published:** 2005-09-15

**Authors:** Peter J Stogios, Gregory S Downs, Jimmy JS Jauhal, Sukhjeen K Nandra, Gilbert G Privé

**Affiliations:** 1 Department of Medical Biophysics, University of Toronto, Toronto, Ontario, M5G 2M9, Canada; 2 Bioinformatics Certificate Program, Seneca College, Toronto, Ontario, M3J 3M6, Canada; 3 Department of Biochemistry, University of Toronto, Toronto, Ontario, M5S 1A8, Canada; 4 Ontario Cancer Institute, 610 University Avenue, Toronto, Ontario, M5G 2M9, Canada

## Abstract

**Background:**

The BTB domain (also known as the POZ domain) is a versatile protein-protein interaction motif that participates in a wide range of cellular functions, including transcriptional regulation, cytoskeleton dynamics, ion channel assembly and gating, and targeting proteins for ubiquitination. Several BTB domain structures have been experimentally determined, revealing a highly conserved core structure.

**Results:**

We surveyed the protein architecture, genomic distribution and sequence conservation of BTB domain proteins in 17 fully sequenced eukaryotes. The BTB domain is typically found as a single copy in proteins that contain only one or two other types of domain, and this defines the BTB-zinc finger (BTB-ZF), BTB-BACK-kelch (BBK), voltage-gated potassium channel T1 (T1-Kv), MATH-BTB, BTB-NPH3 and BTB-BACK-PHR (BBP) families of proteins, among others. In contrast, the Skp1 and ElonginC proteins consist almost exclusively of the core BTB fold. There are numerous lineage-specific expansions of BTB proteins, as seen by the relatively large number of BTB-ZF and BBK proteins in vertebrates, MATH-BTB proteins in *Caenorhabditis elegans*, and BTB-NPH3 proteins in *Arabidopsis thaliana*. Using the structural homology between Skp1 and the PLZF BTB homodimer, we present a model of a BTB-Cul3 SCF-like E3 ubiquitin ligase complex that shows that the BTB dimer or the T1 tetramer is compatible in this complex.

**Conclusion:**

Despite widely divergent sequences, the BTB fold is structurally well conserved. The fold has adapted to several different modes of self-association and interactions with non-BTB proteins.

## Abstract 2

An analysis of the protein architecture, genomic distribution and sequence conservation of BTB domain proteins in 17 fully sequenced eukaryotes reveals a high structural conservation and adaptation to different modes of self-association and interactions with non-BTB proteins.

## Background

The BTB domain (also known as the POZ domain) was originally identified as a conserved motif present in the *Drosophila melanogaster *bric-à-brac, tramtrack and broad complex transcription regulators and in many pox virus zinc finger proteins [1-4]. A variety of functional roles have been identified for the domain, including transcription repression [5,6], cytoskeleton regulation [7-9], tetramerization and gating of ion channels [10,11] and protein ubiquitination/degradation [12-17]. Recently, BTB proteins have been identified in screens for interaction partners of the Cullin (Cul)3 Skp1-Cullin-F-box (SCF)-like E3 ubiquitin ligase complex, with the BTB domain mediating recruitment of the substrate recognition modules to the Cul3 component of the SCF-like complex [18-20]. In most of these functional classes, the BTB domain acts as a protein-protein interaction module that is able to both self-associate and interact with non-BTB proteins.

Several BTB structures have been determined by X-ray crystallography, establishing the structural similarity between different examples of the fold. We use the Structural Classification of Proteins (SCOP) database terminology of 'fold' to describe the set of BTB sequences that are known or predicted to share a secondary structure arrangement and topology, and the term 'family' to describe more highly related sequences that are likely to be functionally similar [21]. Thus, the BTB domain in BTB-zinc finger (ZF), Skp1, ElonginC and voltage-gated potassium channel T1 (T1-Kv) proteins all contain the BTB fold, even though some of these differ in their peripheral secondary structure elements and are involved in different types of protein-protein associations. For example, BTB domains from the BTB-ZF family contain an amino-terminal extension and form homodimers [5,22], whereas the Skp1 proteins contain a family-specific carboxy-terminal extension and occur as single copies in heterotrimeric SCF complexes [23-26]. The ElonginC proteins are also involved in protein degradation pathways, although these proteins consist only of the core BTB fold and are typically less than 20% identical to the Skp1 proteins [27,28]. Finally, T1 domains in T1-Kv proteins consist only of the core fold and associate into homotetramers [11,29]. Thus, while the structures of BTB domains show good conservation in overall tertiary structure, there is little sequence similarity between members of different families. As a result, the BTB fold is a versatile scaffold that participates in a variety of types of family-specific protein-protein interactions.

Given the range of functions, structures and interactions mediated by BTB domains, we undertook a survey of the abundance, protein architecture, conservation and structure of this fold. An earlier study [30] is consistent with many of the results presented here, and we contribute an expanded structure and genome-centric analysis of BTB domain proteins, with an emphasis on the scope of protein-protein interactions in these proteins. Our results should be useful for the structural and functional prediction by analogy for some of the less-well characterized BTB domain families.

## Results and discussion

### BTB fold comparisons

**Figure 1:**
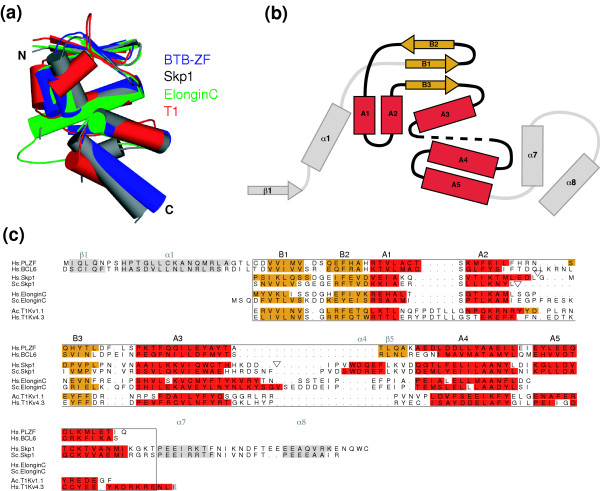
Comparison of structures containing the BTB fold. **(a) **Superposition of the BTB core fold from currently known BTB structures. The BTB core fold (approximately 95 residues) is retained across four sequence families. The BTB-ZF, Skp1, ElonginC and T1 families are represented here by the domains from Protein Data Bank (PDB) structures 1buo:A, 1fqv:B, 1vcb:B, 1t1d:A. **(b) **Schematic of the BTB fold topology. The core elements of the BTB fold are labeled B1 to B3 for the three conserved β-strands, and A1 to A5 for the five α-helices. Many families of BTB proteins are of the 'long form', with an amino-terminal extension of α1 and β1. Skp1 proteins have two additional α-helices at the carboxyl terminus, labeled α7 and α8. The dashed line represents a segment of variable length that is often observed as strand β5 in the long form of the domain, and as an α-helix in Skp1. **(c) **Structure-based multiple sequence alignment of representative BTB domains from each of the BTB-ZF, Skp1, ElonginC and T1 families. The core BTB fold is boxed. Secondary structure is indicated by red shading for α-helices and yellow for β-strands, with the amino- and carboxy-terminal extensions shaded in gray. The low complexity sequences, which are disordered in the Skp1 structures, are indicated by open triangles. See Figure 3 for the PDB codes for the corresponding sequences.

**Figure 2:**
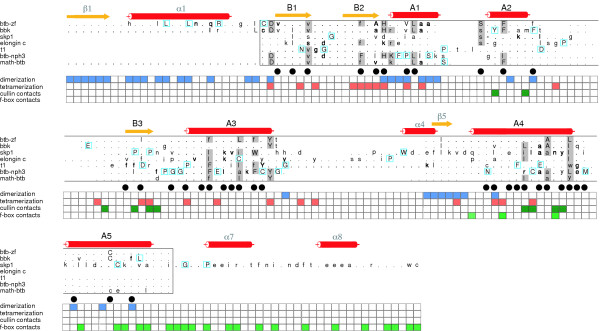
Sequence conservation in BTB domains. The most probable sequences (majority-rule consensus sequences) from each of seven different family-specific hidden Markov models (HMMs) were generated with HMMER hmmemit. Residue positions with a probability score (P(s)) of less than 0.6 are variable and are indicated by dots, residues with 0.6 < P(s) < 0.8 have intermediate levels of sequence conservation and are indicated by lower case letters, and residues with a P(s) > 0.8 are highly conserved and are indicated by capital letters. Gray shading indicates positions that are similar in at least four of the seven families shown, and selected 'signature sequences' that are particular to a specific family are boxed in blue. Gaps are indicated by blank spaces. Residue positions that are buried in the core of the BTB fold are indicated with black circles, and contact sites for four known protein-protein interaction surfaces are shown in the grid below the alignment. The secondary structure elements β1, α1, α4, β5, α7 and α8 occur only in some of the families, and are discussed in the text. Additional Data File 1 includes multiple sequence alignments for these families.

**Figure 3:**
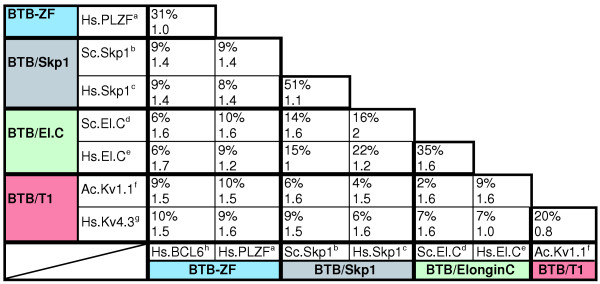
Pairwise sequence and structure comparisons of BTB structures. Cells contain the percentage identity and root mean square deviation (Å) value for each structure pair. Representative structures from the Protein Data Bank are labeled as follows: ^a^1buo:A and 1cs3:A; ^b^1nex:a; ^c^1ldk:D, 1p22:b, 1fqv:B, 1fs1:B, 1fs2:B; ^d^1hv2:a; ^e^1vcb:B, 1lm8:C, 1lqb:B; ^f^1a68:_, 1eod:A, 1eoe:A, 1eof:A, 1t1d:A, 1exb:E (rat Kv1.1); ^g^1s1g:A; ^h^1r28:A, 1r29:A, 1r2b:A. The T1 domains from Kv1.2, Kv3.1 and Kv4.2 were omitted for clarity. El.C, ElonginC. Ac, *Aplysia californica*; Hs, *Homo sapiens*; Sc, *Saccharomyces cerevisiae*.

We began our analysis with a comparison of the solved structures of BTB domains from the Protein Data Bank (PDB) [31], which included examples from BTB-ZF proteins, Skp1, ElonginC and T1 domains (Figures 1, 2, 3). A three-dimensional superposition showed a common region of approximately 95 amino acids consisting of a cluster of 5 α-helices made up in part of two α-helical hairpins (A1/A2 and A4/A5), and capped at one end by a short solvent-exposed three stranded β-sheet (B1/B2/B3; Figure 1). An additional hairpin-like motif consisting of A3 and an extended region links the B1/B2/A1/A2/B3 and A4/A5 segments of the fold. Because of the presence or absence of secondary structural elements in certain examples of the fold, we use the designation A1–A5 for the five conserved α-helices, and B1–B3 for the three common β-strands. We refer to this structure as the core BTB fold. When present, other secondary structure elements are named according to the labels assigned to the original structures. Thus, the BTB-ZF family members the promyelocytic leukemia zinc finger (PLZF) and B-cell lymphoma 6 (BCL6) contain additional amino-terminal elements, which are referred to as β1 and α1, Skp1 protein contains two additional carboxy-terminal helices labeled α7 and α8, ElonginC is missing the A5 terminal helix, and the T1 structures from Kv proteins are formed entirely of the core BTB fold (Figures 1 and 2). Sequence comparisons based on the structure superpositions show less than 10% identity between examples from different families, except for Skp1 and ElonginC, which is in the range of 14% to 22%; however, all structures show remarkable conservation with Root mean square deviation (RMSD) values of 1.0 to 2.0 Å over at least 95 residues (Figure 3). Despite these very low levels of sequence relatedness, 15 positions show significant conservation across all of the structures, and 12 of these correspond to residues that are buried in the monomer core (Figure 2). Most of these highly conserved residues are hydrophobic and are found between B1 and A3, with some examples in A4. In addition to this common set, conserved residues are also found within specific families (Figure 2), and some of these participate in family-specific protein-protein interactions.

**Figure 4:**
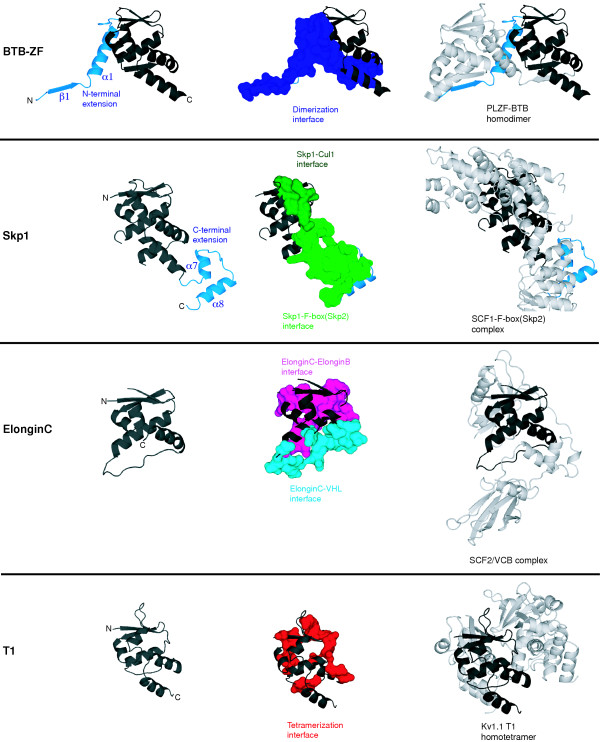
Protein-protein interaction surfaces in BTB domains. Left column: the BTB monomer is shown in the same orientation for each of four structural families with the core fold in black, and the amino- and carboxy-terminal extensions in blue. Middle column: the monomers are shown with the protein-protein interaction surfaces shaded. Right column: the monomers are shown in their protein complexes.

The four known structural classes of BTB domains show different oligomerization or protein-protein interaction states involving different surface-exposed residues (Figures 2 and 4). There is little overlap between the interaction surfaces of the homodimeric, heteromeric and homotetrameric forms of the domain, which are represented here by examples from the BTB-ZF, Skp1/ElonginC and T1 families, respectively. Contacts involving the amino-terminal extensions of the BTB-ZF class and the carboxy-terminal elements of the Skp1 families form a significant fraction of the residues involved in protein-protein interaction in each of those respective systems, but additional contributions from the 95 residue core BTB fold are involved. There are multiple examples of conserved surface-exposed residues that participate in family-specific protein-protein interactions. For example, the B1/B2/B3 sheet is found in all BTB structures and, therefore, is part of the core BTB fold, but participates in very different protein interactions in the T1 homotetramers, the ElonginC/ElonginB and Skp1-Cul1 structures. Inspection of T1 residues in this area shows sequences such as the 'FFDR' motif in B3 have diverged from the other BTB families to become important components of the tetramerization interface [29] (Figure 2). In Skp1, B3 has a distinctive 'PxPN' motif that is involved in Cul1 interactions [24] (Figure 2). Thus, the solvent-exposed surface of the BTB fold is extremely variable between families, forming the basis for the wide range of protein-protein interactions.

The connection between A3 and A4 (drawn as a dashed line in Figure 1b) is variable in length and in structure, and makes key contributions to several different types of protein-protein interactions. The region adopts an extended loop structure in the T1 domain and ElonginC, where it makes important contributions to the homotetramerization and to the von Hippel-Lindau (VHL) interfaces, respectively (Figure 4). In PLZF and BCL6, this segment forms strand β5 and associates with β1 from the partner chain to form a two-stranded antiparallel sheet at the 'floor' of the homodimer [5,22]. In Skp1, this region includes a large disordered segment followed by a unique helix α4, but it is not involved in any protein-protein interactions [23-26].

### Representation of BTB domains in fully sequenced genomes

We searched the Ensembl and Uniprot databases for BTB proteins [32,33]. In order to effectively eliminate redundant sequences and partial fragments, and to reduce sampling bias due to uneven database representation, we limited our search to the known and predicted transcripts from 17 fully sequenced genomes. We carried out HMMER [34] searches with a panel of hidden Markov models (HMMs) describing the four known families of BTB structures. As expected from the low sequence similarities, searches with family-specific HMMs could not retrieve sequences from the other families in a single iteration. For example, the HMM trained on the BTB domains from BTB-ZF proteins could not immediately retrieve sequences from T1-Kv proteins. Additional sequences were added to each of the family-specific HMMs in several cycles, and the results were compiled into final multiple sequence alignments. The retrieved sequences were manually inspected and class-specific HMMs were used to define the start/end sites of specific families of BTB domains. We have assembled this collection of over 2,200 non-redundant BTB domain sequences in a publicly available database [35].

**Figure 5:**
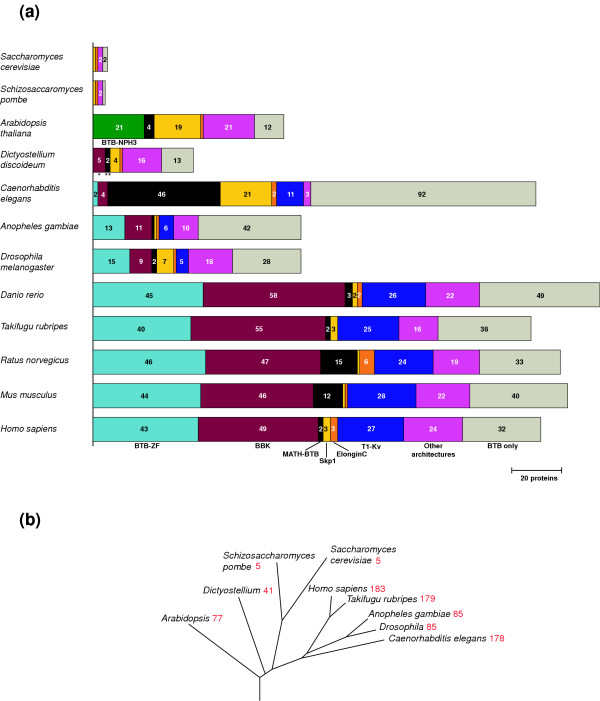
Distribution of BTB proteins in eukarytoic genomes. **(a) **Representation of BTB proteins in selected sequenced genomes. Twelve of the seventeen genomes we searched are represented, showing each type of BTB protein architecture as bar segments. Data for *Apis mellifera*, *Canis familiaris*, *Gallus gallus*, *Pan troglodytes *and *Xenopus tropicalis *are available at [35]. Several lineage-specific expansions are evident: BTB-ZF and BBK proteins in the vertebrates; the MATH-BTB proteins in the worm; the BTB-NPH3 proteins in the plant; the Skp1 proteins in the plant and worm; and the T1 proteins in worm and vertebrates. In the *Dictyostellium discoideum *genome, a single star indicates five BTB-kelch proteins that do not contain the BACK domain, and a double star indicates two MATH-BTB proteins that also contain ankyrin repeats. **(b) **Phylogenetic relationship of analyzed genomes. Adapted from [39]. The total number of BTB proteins is shown above each genome.

In addition to the genome-centric analyses, we searched the NCBI nr database with PSI-BLAST [36,37]. Beginning with the sequence of the BTB domain from the BTB-ZF protein PLZF, T1 sequences were retrieved with e-values below 10 after four PSI-BLAST iterations carried out with a generous inclusion threshold of 0.1, as previously reported [30]. Skp1 and ElonginC sequences could not be retrieved with e-values below 10 starting with BTB-ZF or T1 sequences, even with a PSI-BLAST inclusion threshold of 1.0. Based on searches with representative BTB sequences from each of the major families, BTB sequences were consistently retrieved from eukaryotes and poxviruses, but no examples from bacteria or archaea were found (data not shown), with the remarkable exception of 43 BTB-leucine-rich repeat proteins in the *Parachlamydia*-related endosymbiont UWE25 [38]. In general, plant and animal genomes encode from 70 to 200 distinct BTB domain proteins, while only a handful of examples are found in the unicellular eukaryotes. We identified an intermediate number, 41, in the social amoeba *Dictyostelium discoideum *[39] (Figure 5).

The distribution of BTB families varies widely according to species (Figure 5). The overall number of BTB domain proteins and their family distribution is similar in the mammalian and fish genomes that we considered, with 25 to 50 examples from each of the BTB-ZF, BTB-BACK-kelch (BBK) and T1-Kv families, and another 40 to 50 proteins with other architectures. We expect that this distribution is similar across all vertebrate genomes. The family distribution in the insects (as exemplified by *Drosophila *and *Anopheles*) is generally similar to that of the vertebrates, but with fewer overall examples. In contrast, *Caenorhabditis elegans *contains very few BTB-ZF and BBK proteins, but a large number of meprin and tumor necrosis factor receptor associated factor homology (MATH)-BTB and Skp1 proteins. In *Arabidopsis*, there are 21 BTB-nonphototropic hypocotyl (NPH)3 proteins, which appear to be a plant-specific architecture. Only five and six BTB domain proteins were found in *Saccharomyces cerevisiae *and *Schizosaccharomyces pombe*, respectively.

**Figure 6:**
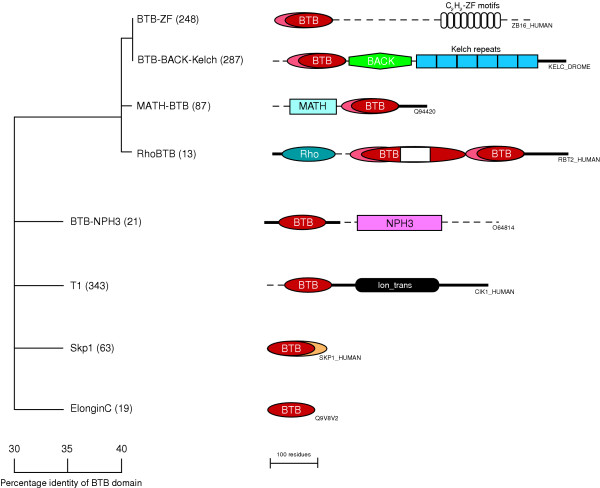
BTB sequence clusters and protein architectures. Family-specific amino- and carboxy-terminal extensions to the core BTB fold are indicated. Regions with no predicted secondary structure are indicated by dashed lines, and ordered regions are indicated with either domain notations or thick solid lines. The Uniprot code for a representative protein is indicated. Clustering by BLASTCLUST was based on the average pairwise sequence identity for all BTB domain sequences from our database, except for the RhoBTB proteins, where only the carboxy-terminal BTB domain was used. Domain names are from Pfam [44].

Based on these observations, the domain most likely underwent domain shuffling followed by lineage-specific expansion (LSE) during speciation events. The most commonly observed architecture across several different families consists of a single amino-terminal BTB domain, a middle linker region, and a characteristic carboxy-terminal domain that is often present as a set of tandem repeats (Figure 6). Along with domain shuffling and domain accretion, LSE is considered one of the major mechanisms of adaptation and generation of novel protein functions in eukaryotes, and is frequently seen in proteins involved in cellular differentiation and in the development of multicellular organisms [40]. For example, both BTB-ZF proteins and Kruppel-associated box (KRAB)-ZF proteins have essential roles in development and tissue differentiation and have undergone LSE in the vertebrate lineage [30,41,42].

### BTB sequence clusters

We attempted to construct a phylogeny based on BTB domain sequences, but we could not consistently cluster the entire collection. Due to the very low levels of sequence similarity between some of the families (Figure 3), we were unable to support phylogenies with significant bootstrap values despite many attempts with several different approaches and algorithms, including distance, maximum parsimony or maximum likelihood methods.

We eventually turned to BLASTCLUST as a more appropriate tool to subdivide this highly divergent set of sequences [37] (Figure 6). BTB domain sequence/structure families correlate with the protein architectures, and the BTB-NPH3, T1, Skp1 and ElonginC families could be distinguished at an identity threshold of 30% with this method. Domain sequences from BTB-ZF, BBK, MATH-BTB and RhoBTB proteins formed distinct clusters only at higher cutoffs, and are thus more closely related (Figure 6). The BTB domain sequences from vertebrate BTB-ZF and BBK proteins are more closely related, and cannot be separated by BLASTCLUST.

### Long form of the BTB domain

The majority of BTB domains from the BTB-ZF, BBK, MATH-BTB, RhoBTB and BTB-basic leucine Zipper (bZip) proteins contain a conserved region amino-terminal to the core region, which likely forms a β1 and α1 structure as seen in PLZF [22,43] and BCL6 [5]. We refer to this as the 'long form' of the BTB domain, which has a total size of approximately 120 residues. Note that many of the protein domain databases, such as Pfam [44], SMART [45] and Interpro [46], recognize only the 95 residue core BTB fold, and do not detect all of these additional elements, even though at least half of the metazoan BTB domains correspond to the long form. The long form BTB domain sequences also are more highly related to each other than to the BTB-NPH3, T1, Skp1 and ElonginC families, as based on the BLASTCLUST analysis (Figure 6). These groupings were consistently observed even when only the residues from the core fold were included in the analysis, and so the sequence clustering is not simply due to the presence or absence of the amino-terminal elements. We predict that most long form BTB domains are dimeric, and that several of these associate into higher order assemblies via inter-dimer sheets involving β1, as discussed below.

### The BTB-ZF proteins

BTB-ZF proteins are also known as the POK (POZ and Krüppel zinc finger) proteins [47]. Many members of this large family have been characterized as important transcriptional factors, and several are implicated in development and cancer, most notably BCL6 [48,49], leukemia/lymphoma related factor (LRF)/Pokemon [47], PLZF [50], hypermethylated in cancer (HIC)1 [51,52] and Myc interacting zinc finger (MIZ)1 [53].

In the BTB-ZF setting, the domain mediates dimerization, as shown by crystallographic studies of the BTB domains of PLZF [22] and BCL6 [5]. This is confirmed in numerous solution studies [5,22,43,54-56]. An important component of the hydrophobic dimerization interface in PLZF and BCL6 is the association of the long form elements β1 and α1 from one monomer with the core structure of the second monomer. The dimerization interface has two components: an inter-molecular antiparallel β-sheet formed between β1 from one monomer and β5 of the other monomer; and the packing of α1 from one monomer against α1 and the A1/A2 helical hairpin from the other monomer. The strand-exchanged amino terminus is likely to have arisen from a domain swapping mechanism [57]. We believe that most BTB domains from human BTB-ZF proteins can dimerize, because 34 of these 43 domains are predicted to contain all of the necessary structural elements in the swapped interface including β1, α1 and β5 (Additional data file 1). As well, many highly conserved residues are found in predicted dimer interface positions [22]. Nine human BTB-ZF proteins lack β1, and thus cannot form the β1–β5 interchain antiparallel sheet, and we expect that these domains are also dimeric due to the presence of α1 and the conservation of interface residues. In PLZF and BCL6, the BTB domain forms obligate homodimers [5,22], and disruption of the dimer interface results in unfoldfed, non-functional protein [6].

In nearly all BTB-ZF proteins, the long form BTB domain is at or very near the amino terminus of the protein, and the Krüppel-type C_2_H_2 _zinc fingers are found towards the carboxyl terminus of the protein. These two regions are connected by a long (100–375 residue) linker segment (Figure 6). Sequence conservation is largely restricted to the BTB domain and the carboxy-terminal ZF region, as exemplified by BCL6 from human and zebrafish, which are 78%, 37% and 85% identical across the BTB, linker and ZF regions, respectively. The linker region frequently contains low complexity sequence and is predicted to be unstructured in most cases. Except for proteins that are highly related over their full lengths, the linker regions do not identify significant matches in sequence searches of the NCBI nr set. This architecture suggests a model in which the dimeric BTB domain connects the DNA binding regions from each chain via long, mostly unstructured tethers. Thus, we expect that the DNA binding ZF domains can bind two promoter sites, but that the exact spacing and orientation of these sites is not critical, as long as they are within a certain limiting distance. The linker is not without function, however, as it interacts with accessory proteins that take part in chromatin remodeling and transcription repression, such as the BCL6-mSin3A and PLZF-ETO interactions [6,58].

The BTB domains from some BTB-ZF proteins can mediate higher order self-association [59-62], and the formation of BTB oligomers in the BTB-ZF proteins has important implications for the recognition of multiple recognition sequences on target genes. In *Drosophila *GAGA factor (GAF), oligomerization of BTB transcription factors is thought to be mechanistically important in regulating the transcriptional activity of chromatin [61,62], and the BTB domain is essential in co-operative binding to DNA sites containing multiple GA target sites [62]. Several other BTB transcription factors also bind to multiple sites [52,60,63]. The formation of chains of BTB dimers involving the β1/β5 'lower sheet' has been observed in two different crystal forms of the PLZF BTB domain [22,43], although the significance of this is unclear as BTB dimer-dimer associations are very weak and are not observed in solution under normal conditions (unpublished results and [43]). Higher-order association could be a property of a subset of BTB domains, with GAF-BTB representing domains that have a strong propensity for polymerization, whereas in cases such as PLZF-BTB, the self-association of dimers is observed only at very high local protein concentrations, such as those required for crystal formation. Interestingly, many *Drosophila *BTB domains have characteristic hydrophobic sequences in the β1 and β5 regions [1]. In many of these, the β1 region contains at least three large, hydrophobic residues in a characteristic [FY]×[ILV]×[WY][DN][DN][FHWY] sequence that is not present in BTB-ZF proteins from other species. This conserved segment has high β-strand propensity, consistent with the presence of interchain β1 contacts across dimers. Exposed hydrophobic residues in this sheet region may drive strong dimer-dimer associations in these *Drosophila *BTB-ZF proteins, an idea that is supported by modeling studies [64].

Heteromeric BTB-BTB associations have been described between certain pairs of BTB domains from this family, including PLZF and Fanconi anemia zinc finger (FAZF) [65], and between BCL6 and BCL6 associated zinc finger (BAZF) [66]. Heteromer formation in BTB transcription factors may be a mechanism for targeting these proteins to particular regulatory elements by combining different chain-associated DNA binding domains in order to generate distinct DNA recognition specificities [67], as seen in retinoic acid receptor/retinoid X receptor transcription factors [68].

In addition to the architectural roles resulting from BTB-BTB associations, many BTB domains in this family interact with non-BTB proteins, and this effect is central to their function in transcriptional regulation. For example, BCL6 is able to associate directly with nuclear co-repressor proteins such as nuclear co-repressor (NCoR), silencing mediator for retinoid and thyroid hormone receptors (SMRT) and mSin3a [5,58,69-73]. A 17 residue region of the SMRT co-repressor binds directly with the BCL6 BTB domain in a 2:2 stoichiometric ratio in a complex that requires a BCL6 BTB dimer [5]. This peptide is an inhibitor of full-length SMRT, and reverses the repressive activities of BCL6 *in vivo *[48]. Remarkably, the interaction with this peptide appears to be specific to the BCL6 BTB domain, and there is no significant sequence conservation in the BCL6 peptide binding groove relative to other human BTB-ZF proteins. In these other proteins, this groove may be a site for as yet uncharacterized BTB-peptide or BTB-protein interactions.

In all organisms studied, BTB domains from BTB-ZF proteins show high conservation of the residues Asp35 and Arg/Lys49 (PLZF numbering; Additional data file 1). These residues are found in a 'charged pocket' in the BTB structures of PLZF and BCL6, and have been shown to be important in transcriptional repression [6,74]. The structure of the BCL6-BTB-SMRT co-repressor complex, however, did not show interactions between this region and the co-repressor [5]. Mutation of Asp35 and Arg49 disrupts the proper folding of PLZF [6], and these residues are thus important for the structural integrity of the domain. Interestingly, Asp35 and Arg/Lys49 are also conserved in the BTB domains from BBK, MATH-BTB and BTB-NPH3 proteins (Figure 2 and Additional data file 1).

### The BBK proteins

Many members of this widely represented family of proteins are implicated in the stability and dynamics of actin filaments [75-78]. With few exceptions, all of the 515 BTB-kelch proteins in our database also contain the BTB and carboxy-terminal kelch (BACK) domain. These BBK proteins are composed of a long-form BTB domain, the 130 residue BACK domain [79], and a carboxy-terminal region containing four to seven kelch motifs [80-82]. Most BBK proteins have a region of approximately 25 residues that precede the BTB domain, unlike BTB-ZF proteins where BTB is positioned much closer to the amino terminus (Figure 6; Additional data file 1). We predict that this amino-terminal region in the BBK proteins is unstructured, although it is shown to have a functional role in some proteins [75]. Notably, the distribution of BBK proteins parallels that of the BTB-ZF proteins across genomes. We did not find BBK proteins in *Arabidopsis thaliana *or in the yeasts.

The sequences of BTB domains from BBK proteins are most closely related to those from BTB-ZF proteins (Figure 6), suggesting that they adopt similar structures. Indeed, BTB domains from BBK proteins have been shown to mediate dimerization [75,83,84] and have conserved residues at positions equivalent to those at the dimer interface in BTB-ZF proteins (Additional data file 1). There are reports of BTB-mediated oligomerization in BBK proteins, consistent with the role of some these proteins as organizers of actin filaments [75,77,84]. Because most of the BTB sequences from BBK proteins are predicted to contain the β1, α1 and β5 long form elements, oligomerization of these proteins may occur via dimer-dimer associations involving the β1 sheet, as proposed for the BTB-ZF proteins. There are, however, no strongly characteristic sequences or enrichment of hydrophobic residues in the β1 region.

In Pfam, the POZ domain superfamily (Pfam Clan CL0033) includes BACK, BTB, Skp1 and K_tetra (T1) sequences [44]. The known structures of BTB, Skp1 and T1 domains show the conserved BTB fold, and the inclusion of the BACK domain in this Pfam Clan suggests that the BACK domain also adopts this fold. Secondary structure predictions for BTB, Skp1 or T1 domain sequences, however, consistently reflect the known mixed α/β content of the BTB fold, whereas the BACK domain is predicted to contain only α-helices [79]. Further clarification of this issue will require the experimental determination of the structure of the BACK domain.

### Skp1

Skp1 is a critical component of Cul1-based SCF complex, and forms the structural link between Cul1 and substrate recognition proteins [85-87]. Skp1 proteins are only distantly related to other BTB families (Figures 3 and 6), and are composed of the core BTB fold with two additional carboxy-terminal helices. These latter helices form the critical binding surface for the F-box region of substrate-recognition proteins. Many Skp1 sequences have low complexity insertions after A3, which are disordered in several crystal structures, followed by helix α4, which is unique to this family [23-26] (Figures 1 and 2). Skp1 proteins are found in all organisms studied, with significant expansions in *C. elegans *and *A. thaliana *(Figure 5). Interestingly, the Cul1-interacting surface of Skp1 does not overlap with the dimerization surface seen in BTB-ZF structures, and is mostly separate from the tetramerization surface in the T1 domains (Figure 2; Additional data file 1). Therefore, a unique surface of the BTB fold in the Skp1 proteins has adapted to mediate interactions with Cul1.

### ElonginC

ElonginC is an essential component of Cul2-based SCF-like complexes, also known as VCB (for pVHL, ElonginC, ElonginB) or ECS (for ElonginC, Cul2, SOCS-box) E3 ligase [88,89]. This protein serves as an adaptor between ElonginB and the VHL tumor suppressor protein, which interacts with hypoxia inducible factor (HIF)-1α and targets it for degradation [89-92]. In any given organism, the sequence identity between ElonginC and Skp1 is approximately 30% or less, but these proteins are nonetheless more closely related to each other than to other BTB sequences (Figure 3). The structure of ElonginC showed that it is composed entirely of the core BTB fold, but lacks the terminal A5 helix [27,28,93,94]. We found ElonginC proteins in all organisms studied (Figure 5). Like Skp1, ElonginC is significantly similar to other BTB sequence classes only in the buried positions of the monomer core (Figure 2). A β-strand in the A3/A4 connecting region participates in the ElonginC-VHL interaction, and the sequence in this region is characteristic of ElonginC [28].

### The T1 domain in Kv channels

The T1 domain from voltage-gated potassium channels modulates channel gating and assembly [10,29,95]. This domain is a distant homolog to all other BTB domains, and segregates into a unique cluster at less than 30% sequence identity with BLASTCLUST. The T1 domain is found in a large number of voltage-gated potassium channel proteins in all metazoan genomes surveyed (Figure 5). T1 sequences have been classified according to their sequence similarity into nine Kv families (Kv1 through Kv9) [96,97]. The full-length protein sequences are composed of a disordered amino-terminal region, the T1 domain, a transmembrane ion transduction domain (Pfam PF00520), and a long carboxy-terminal region with some predicted secondary structure (Figure 6).

Structurally, the T1 domain is composed of the core BTB fold without any amino- or carboxy-terminal extensions (Figures 1 and 2; Additional data file 1). The T1 domain mediates homo-tetramerization in numerous crystal structures [11,29,98,99]. Despite the very low levels of sequence similarity to the other BTB domain families, several of the characteristic buried residues are conserved (Figure 2). It is striking that most of the residues found in the polar tetramerization contact surface in the T1 structures do not overlap with those residues involved in dimerization in the BTB-ZF structures. Of the 24 residues that are found in the T1 tetramer surface, only 6 are common to the BTB-ZF dimer interface (Figure 2). Thus, a unique set of residues has evolved in the T1 domain to mediate tetramerization.

### The MATH-BTB proteins

A large expansion of MATH-BTB proteins occurred in *C. elegans*, where 46 of 178 total BTB proteins belong to this family, whereas other genomes contain many fewer of these proteins (Figure 5). MATH proteins as a whole are largely expanded in *C. elegans*, with 95 examples present in the Pfam database [44]. The MATH domain is thought to be a substrate recognition module in Cul3-based SCF-like complexes [15,16].

MATH-BTB proteins differ from most other BTB families in that the BTB domain is found carboxy-terminal to the partner domain. Typically, there are an additional 75 to 100 amino acids following the BTB domain that are likely to be structured and rich in α-helices (Additional data file 1). In contrast to the BTB-ZF proteins, but similar to the BBK proteins, MATH-BTB sequences are highly conserved across the full lengths of the proteins. As a result of this conservation, phylogenetic clustering of the full-length protein sequences can be done with reasonable bootstrap values and shows a clear demarcation between proteins from *C. elegans *and those from all other species (data not shown). The domain in the *C. elegans *proteins lacks several BTB signature sequences, such as the 'AH[RK]XVLAA' signature in the B2-A1 region seen in many other long form BTB families (Figure 2). The majority of MATH-BTB proteins from all organisms are predicted to contain the long form elements β1, α1 and β5 (Additional data file 1) and we predict that these BTB domains are dimeric. Indeed, biochemical and biological evidence suggest that BTB-mediated dimerization of the MATH-BTB protein maternal effect lethal (MEL)-26 is required for its function [15,100].

### The BTB-NPH3 proteins

Another large expansion is found in *Arabidopsis*, which contains 21 BTB-NPH3 proteins, or over 25% of the BTB proteins in this genome. BTB-NPH3 proteins are not found in any of the other genomes that we considered, and could represent a plant-specific adaptation of the BTB domain. BTB-NPH3 proteins are involved in phototropism in *A. thaliana *and are thought to be adaptor proteins that bring together components of a signal transduction pathway initiated by the light-activated serine/threonine kinase NPH1 [101,102]. Heteromerization of BTB-NPH3 proteins have been observed, and the BTB domains of root phototropism (RPT)2 and NPH3 have been shown to interact [101,102]. In addition, the BTB domain from RPT2 can interact with a region of phototropin 1 that contains light, oxygen and voltage sensing (LOV) protein-protein interaction domains [103]. These proteins consist of an amino-terminal BTB domain and an NPH3 domain (Figure 6). The BTB domains in this family are only distantly related to other examples of the fold, and appear to have two leading β-strands in a region preceding the core fold, with an additional β-strand between A1 and A2 (Additional data file 1).

### BTB-bZip proteins

Each of the vertebrate genomes considered here contain genes for two BTB-bZip proteins, named BTB and CNC homology (BACH)1 and BACH2 [104,105], except for *Danio rerio*, which has three. These proteins are transcription factors and most closely resemble the BTB-ZF proteins in terms of the BTB sequence and overall protein architecture. The proteins consist of a long form BTB domain, a central region of approximately 400 residues, and a carboxy-terminal basic leucine zipper region (Figure 6). The close similarity of the BTB sequences between the BTB-ZF and BTB-bZip proteins suggest that these domains are likely to be similar in structure. Notably, the long form elements and β5 are predicted, and dimerization residues are similar to the ZF class (data not shown). Accordingly, the BACH proteins have been shown to dimerize and oligomerize in a BTB-dependent manner [63]. bZip domains themselves are known to dimerize and, interestingly, the majority of bZip-containing proteins (550 of 738 Pfam bZip_1 domain) contain no other identified domains in the full-length protein [44]. Therefore, the domain composition and sequence properties of BTB-bZip proteins are unusual in the context of all bZip proteins, but are compatible with dimeric, and most likely oligomeric, BTB transcription factors.

### The RhoBTB proteins

The Ras homology (Rho)BTB proteins have an unusual architecture, and contain a Rho GTPase domain near the amino terminus, two tandem long form BTB domains, and an approximately 100 residue carboxy-terminal tail with predicted α-helical content (Figure 6). These proteins are highly conserved across their full-lengths, and three examples (RhoBTB1, RhoBTB2/DBC2, RhoBTB3) are found in each of the vertebrates included in this study [106-108]. One RhoBTB protein is also present in the insects and in *Dictyostelium *[107]. The first BTB domain of human RhoBTB2 has been shown to interact with Cul3 [13] and contains a large 115 residue insertion between A2 and B3, while the second domain is more typical and most closely resembles BTB domains from BBK proteins. The tandem domains are immediately adjacent and may form an intramolecular dimer.

Mutations have been identified in lung cancer patients that do not disrupt the RhoBTB2-Cul3 interaction [13], and these map to regions outside of the predicted Cul3-interacting region (see below). We predict, however, that the Y284D cancer mutation is found in the dimerization interface of the first BTB domain and prevents the proper folding of the domain. This would be analogous to mutants in the dimer interface of PLZF that abrogate function by affecting the folding of the domain [6]. The PLZF and BCL6 BTB domains are obligate dimers, and cannot fold as stable monomers (unpublished observation and [43]).

### The BTB-BACK-PHR (BBP) proteins

Sequence analysis on proteins with the BTB-BACK architecture but no kelch repeats revealed the presence of a conserved carboxy-terminal region of approximately 170 residues. This region in the BTBD1 and BTBD2 proteins has sequence similarity with human protein associated with myc (PAM; NCBI accession number AAC39928), *Drosophila *highwire (AAF76150) and *C. elegans *regulator of presynaptic morphology (RPM-1; NP_505267.1) and has been called the 'PHR-like' region (Pfam accession PF08005). It has been shown to interact with topoisomerase 1 [109].

Searches with various PHR domain sequences against the Pfam, Prodom and SMART databases identified only automatically generated alignments, and BLAST searches against the PDB did not reveal any significant hits. The domain does not contain extended regions of disorder, and secondary structure predictions suggest that the PHR domain is an all-β fold. Despite the lack of a strongly repeating sequence motif, the PHR may represent a novel type of β-propeller structure, by analogy with the BBK proteins. Using HMM searches, we found from four to seven examples of BTB-BACK-PHR (BBP) proteins in the metazoan genomes, including mammalian BTBD1, BTBD2, BTBD3 and BTBD6. We adopted the name 'PHR domain' for this motif and it has been added to the Pfam database as accession PF08005.

### The BTB-ankyrin proteins

Ankyrin repeats are common protein-protein interaction motifs that are found in proteins of very diverse function, such as transcription regulators, ion transporters and signal transduction proteins [110,111]. We found examples of BTB-ankyrin proteins in each species that we considered, although, unlike other BTB domain families, these proteins do not fit a single canonical arrangement. For example, some BTB-ankyrin proteins are composed of an amino-terminal BTB domain, a central helical region, 19 ankyrin repeats and a carboxy-terminal FYVE domain (a domain originally found in Fab1, YOTB, Vac1, and EEA1 proteins; Pfam accession PF01363), whereas other examples contain two ankyrin repeats followed by a linker region, two tandem BTB domains, and a 300 residue carboxy-terminal helical region. The three BTB-ankyrin proteins from *S. pombe *(Btb1p, Btb2p, Btb3p) are components of a SCF-like ubiquitin ligase complex and interact with Pcu3p, a Cul3 homolog [17]. Both BTB domains of Btb3p are necessary for this interaction. The BTB sequences from these proteins are only distantly related to other BTB domains, and we thus cannot reliably predict the nature of their interaction surfaces.

### BTB proteins with no other identified domain

A significant number of BTB proteins do not contain other identified sequence motifs (Figure 5). Excluding the Skp1 and ElonginC proteins, 52% of the *C. elegans *BTB proteins, but only 17% of the human proteins, belong to this family. There may be additional domains in some of these proteins that have yet to be identified.

### BTB domains in cullin complexes

Several members of the BTB families described here have been found to interact with Cul3-based SCF-like complexes including BTB-ZF [14], BBK [12,14,112], MATH-BTB [14-16], RhoBTB [13], BTB-ankyrin [17], BTB-only [14,17] and T1-Kv [16] proteins. The roles of Skp1 and ElonginC as integral components of SCF and VCB complexes, respectively, have long been established [86,113]. In SCF complexes, F-box proteins such as Cdc4 form precise complexes with Skp1 helices α7 and α8 via their F-box, thus positioning their ligand-binding carboxy-terminal WD40 β-propeller domain such that bound substrate is ubiquinated by the E3 ligase [25,26].

Nine of the 49 human BBK proteins have been identified as components of Cul3-based SCF-like complexes [12,14] and, in several cases, the BTB domain is necessary and sufficient for interaction with Cul3. We propose that the BBK proteins are structurally analogous to the two-chain Skp1/Fbox or ElonginC/SOCS box complexes [79]. In these cases, the central BACK domain would serve to position the carboxy-terminal β-propeller kelch repeats for substrate recognition [114]. We expect a similar situation in the BBP proteins, where the PHR domain would act at the substrate recognition module.

BTB domains of 5 of the 46 MATH-BTB proteins from *C. elegans *have been shown to interact with Cul3. As in the BBK proteins, the MATH-BTB proteins are conserved over much of their entire length, and are likely to be internally rigid. In this scenario, the substrate-recognizing MATH domain is found amino-terminal to the BTB domain, but since the amino and carboxyl termini are very close to each other in the long form BTB domain dimer [5,22], the MATH domain in these proteins may occupy a similar spatial position relative to the BTB dimer as the BACK-kelch region of BBK proteins.

Some BTB-ZF proteins, including PLZF, have also been shown to bind to Cul3, presumably in a BTB-dependent mode [14]. The role of these proteins in Cul3-based SCF-like complexes pose a puzzle, as we do not expect that downstream ZF domains maintain a fixed orientation relative to the BTB domain due to the structurally disordered central region. Further work will be required to understand the structure and function of BTB-ZF proteins in SCF-like complexes.

### A model of the ubiquitin-E2-Cul3-Rbx1-BBK complex

**Figure 7:**
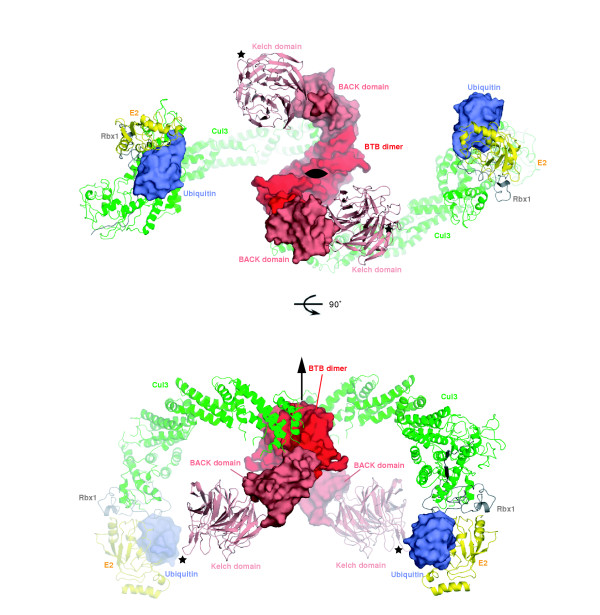
Structural model of the ubiquitin-E2-Cul3-Rbx1-BBK complex. The complex forms a dimer by the self-association of the BTB domain in the BBK protein. The approximate position of the two-fold axis is indicated. Each full-length BBK protein is shown in red, with the BTB dimer shown in the darkest shading in surface representation, the two BACK domains in pink surface, and the two Kelch β-propellers shown in pink cartoon representation. The Cul3 homology model is shown in green cartoon representation, Rbx1 is in gray cartoon representation, E2 Ubch7 is in yellow cartoon representation, and ubiquitin is shown as a blue surface. Stars indicate the position associated with substrate binding [114]. Depth cuing is used to indicate distances in the plane of the page, such that the diffuse colors are most distant to the viewer than the intense colors.

To aid in understanding the role of the BTB domain in the SCF-like complex, we generated a structural model of a BBK protein dimerized via its BTB domain in a complex with Cul3, Rbx1, E2 and ubiquitin (Figure 7). Three different structures of Skp1 complexes are known [24-26], including a Cul1-Skp1 complex [24]. We generated a homology model of human Cul3 based on the structure of Cul1, and placed the PLZF BTB dimer by superposing one chain of the dimer with Skp1. Residues in Skp1 that interact with Cul1 are found at positions that do not involve the dimer interface residues in PLZF (Figures 2 and 4). The BTB domain from the BTB-ZF, BBK and MATH-BTB and BTB-bZip families are closely related (Figure 6) and contain mostly the long form of the domain, as discussed above. We predict these to form obligate dimers, similar to those observed in PLZF and BCL6 [5,22,55]. Proteins from each of these families have been shown to interact with Cul3; therefore, it is reasonable to postulate that these BTB domains drive the dimerization of Cul3 complexes. Indeed, dimerization of adaptor proteins is known to occur [115]. The resulting model is similar to the model presented for the ubiquitin-E2-SCF(Cdc4) [26] and E2-SCF^β-TrcP1 ^complexes [25], except that two ligand-binding kelch/WD40 domains and two E2-ubiquitins localize to the same face of the dimeric complex. In each BBK protein, the BACK domain is between the amino-terminal BTB domain and the carboxy-terminal ligand binding domain, and is likely to be important for positioning the substrate in the complex. A more precise model for a dimeric Cul3-based E3 ligase complex will require the structure of the BACK domain.

Interestingly, some T1-Kv proteins interact with Cul3 [16], and an equivalent analysis allows the placement of the T1 tetramer into a model of the SCF-like complex (data not shown), although the tetramerization interface is not fully separate from the putative Cul3 interface (Figure 2). Minor structural adjustments that are not evident from the homology modeling may be required in these cases.

## Conclusion

This study illustrates the diversity in the abundance, distribution, protein architecture and sequence characteristics of BTB proteins in 17 eukaryotic genomes. We surveyed public databases and fully sequenced genomes and identified several lineage-specific expansions. The BTB domain is found in a wide variety of proteins, but it most often occurs as a single copy at or near the protein amino terminus. Residues exposed at the surface of the BTB fold are highly variable across sequence families, reflecting the large number of self-association and protein-protein interaction states seen in solved BTB structures. Most BTB-ZF, BBK and MATH-BTB proteins contain a long form of the domain that has an additional conserved amino-terminal region, and these are predicted to form stable dimers. In at least some of the BTB transcription factors, BTB dimers are required for interaction with co-repressor peptides, and possibly for higher order self-association. Based on structural superpositions, we show that the Cul3 interaction surface on many BTB proteins does not overlap with the dimerization interface and, therefore, these BTB proteins may drive the dimerization of Cul3-based E3 ligase complexes.

## Materials and methods

### Structure alignment

Twenty-five entries comprising nine unique BTB structures were retrieved from the PDB with DALI [116], CE [117] and VAST [118] structure superposition searches. Structural superpositions and sequence alignments were generated with CE, SwissPDBViewer [119] and by manual inspection and adjustments. RMSD values were calculated using SwissPDBViewer, and molecular representations were generated with Pymol [120].

### Generation of HMMs

A panel of HMMs describing various families of BTB proteins were trained on structure-guided, manually inspected sequence alignments of BTB domains from the BTB-ZF, BBK, MATH-BTB, T1, Skp1, ElonginC and BTB-NPH3 families. HMMs were matured by iteratively building the results from multiple rounds of sequence search, alignment and training. HMM training and calibration were done with hmmbuild and hmmcalibrate, using default options, from HMMER 2.3.2 [34]. Family-specific HMMs, including long-form BTB domain HMMs, are available at [35].

### Genome collection and sequence searches

All peptides from the translations of all known and predicted transcripts in the genomes of *Anopheles gambiae*, *Apis mellifera*, *Caenorhabditis elegans*, *Canis familiaris*, *Danio rerio*, *Drosophila melanogaster*, *Gallus gallus*, *Homo sapiens*, *Mus musculus*, *Pan troglodytes*, *Rattus norvegicus*, *Takifugu rubripes *and *Xenopus tropicalus *were retrieved from the latest version of Ensembl [32]. *Arabidsopsis thaliana*, *Saccharomyces cerevisiae *and *Schizosaccharomyces pombe *protein sequences were retrieved from Uniprot [46]. *Dictyostelium discoideum *protein sequences were retrieved from Dictybase ('primary features') [121]. Proteins containing BTB domains were identified using hmmsearch from the HMMER package [34], with an e-value cutoff of 10, using our panel of HMMs. BTB domains scoring in the e-value range 0.1 to 10 were manually inspected. Peptide sequences, identifiers, names and aliases, domain boundaries of the non-BTB domains (from Pfam annotations [44] included in the Ensembl peptide features) were stored in an Oracle database.

### Secondary structure prediction

Secondary structure predictions on representative members of each BTB family were completed using the PredictProtein server and the PHD algorithm [122]. Scores above 8 over at least 4 consecutive residues were considered valid predictions. Low complexity regions were detected using SEG, at the PredictProtein server. Regions of inherent sequence disorder were detected using the PONDR [123] and DISOPRED [124] servers.

### Sequence alignment, clustering and most probable sequence detection

Family-specific HMMs were utilized to generate multiple sequence alignments, which were then merged into larger alignments for clustering. Phylogenetic clustering was attempted with the distance, maximum parsimony and maximum likelihood algorithms in the PAUP*4.0 [125], MEGA 2.0 [126], Clustal [127] and PHYLIP 3.63 [128] software packages. The most probable sequences shown in Figure 2 were retrieved using the hmmemit program from the HMMER package [34]. The source code for hmmemit was modified to emit consensus sequences with a probability of 0.4, 0.6 and 0.8 from HMMs for each of the seven families shown in Figure 2.

### Structure modeling

A model of the ubiquitin-E2-Cul3-Rbx1-BBK complex was generated following the approach used in making the ubiquitin-E2-SCF(Cdc4) model [26]. The BBK model was made from a composite of the Skp1 and F-box proteins from the Skp1/Cdc4 [26] and Cul1-Rbx1-Skp1-Skp2 complexes [24], in which one chain of the PLZF BTB dimer [22] was substituted for Skp1, and the BACK domain was assumed to adopt the same structure as Skp1 helices α6 and α7 and the F-box and helical linker regions. The Keap1 kelch domain [114] was used to replace the β-propellers of the Cdc4 WD40 domain. Cul1 was replaced by a homology model of Cul3 that was generated using the 3D-PSSM server [129]. The E2 enzyme Ubch7 was positioned using a superposition of the RING domains from Rbx1 and c-Cbl from the c-Cbl-Ubch7 complex [130], and the placement of ubiquitin was achieved by superposition of the two E2 enzymes Ubch7 and E2-24 from the structure of the E2-24-ubiquitin complex [131].

## Additional data files

The following additional data are available with the online version of this paper. Additional data file 1 contains multiple sequence alignment of BTB domains from BTB-ZF, BBK, Skp1, T1-Kv, MATH-BTB and BTB-NPH3 proteins.

## Supplementary Material

Additional data file 1 Multiple sequence alignment of BTB domains from BTB-ZF, BBK, Skp1, T1-Kv, MATH-BTB and BTB-NPH3 proteins. Click here for file
