# Supplementary material for: Sequence and structural analysis of BTB domain proteins
Source: Genome Biol. 2005 Sep 15;6(10):R82. doi: 10.1186/gb-2005-6-10-r82 (PMC1257465; doi:10.1186/gb-2005-6-10-r82)

Multiple sequence alignments of BTB domains from BTB-ZF, BBK, Skp1, T1-Kv, MATH-BTB and BTB-NPH3 proteins.

These alignments include selected BTB domains from the major LSE's of BTB genes. LSE's were defined as expansions of more than 20 BTB genes from a specific domain architecture class in a specific phylogenetic taxa. The organism abbreviation (Hs = *Homo sapiens*, Mm = *Mus musculus*, Rn = *Rattus norvegicus*, Fr = *Fugu rubripes*, Dr = *Danio rerio*, Dm = *Drosophila melanogaster*, Ag = *Anopheles gambiae*, Ce = *Caenorhabditis elegans*, At = *Arabidopsis thaliana*, Sc = *Saccharomyces cerevisiae*, Sp = *Schizosaccharomyces pombe*), common protein name and Uniprot identifiers for each protein are indicated. The BTB domain region (with or without N- or C-terminal extensions) of the full-length protein is boxed. Black shading indicates residues that are 90% identical; grey shading with white text indicates 75% identity; light grey shading with black text indicates 50% identity. Residues that are known (in the BTB-ZF, Skp1, T1 families) or predicted (in the BBK, MATH-BTB, BTB-NPH3 families) to be buried in each structure are indicated by black circles: a filled black circle indicates 100% similarity and a half-filled black circle indicates 75% similarity across the family. The grid below each alignment shows the known or putative involvement of each residue in protein-protein interactions: known dimerization contacts from BTB-ZF structures (blue); known tetramerization contacts from the T1 structure from Kv1.1 (red); cullin-contacting residues inferred by analogy from the Skp1-Cul1 structures (dark green); F-box-

contacting residues in Skp1 (light green). Consensus secondary structure predictions by PHD are shown below the alignments.

A: Multiple sequence alignment of BTB domains from BTB-ZF proteins. The N-terminal methionine is indicated in bold.

B: Multiple sequence alignment of BTB domains from BBK proteins. The N-terminal methionine is indicated in bold, and BACK domain residues are shaded green.

C: Multiple sequence alignment of Skp1 proteins. The N-terminal methionine is bold type, the C-terminal residue is italicized. Note that the secondary structure naming differs from other BTB domains because Skp1 lacks the N-terminal extension to the core BTB fold. Triangles indicate insertions of 27 and 30 residues in CB34_YEAST and O77430, respectively.

D: Multiple sequence alignment of T1 domains. The N-terminal methionine is bold type, and the region immediately trailing the BTB domain and the transmembrane (TM) domain (shaded purple) is indicated. Note that the secondary structure naming differs from other BTB domains as T1 lacks the N-terminal extension to the core BTB fold.

E: Multiple sequence alignment of BTB domains from MATH-BTB proteins. The MATH domain is shaded cyan, consensus predicted secondary structure C-terminal to the BTB domain is shown in pink, and C-terminal residues are italicized.

F: Multiple sequence alignment of BTB domains from BTB-NPH3 proteins. The N-terminal methionine is bold type, and the linker (with two predicted helices and a poorly conserved region) and NPH3 regions are shown.

A. BTB domains from BTB-ZF proteins

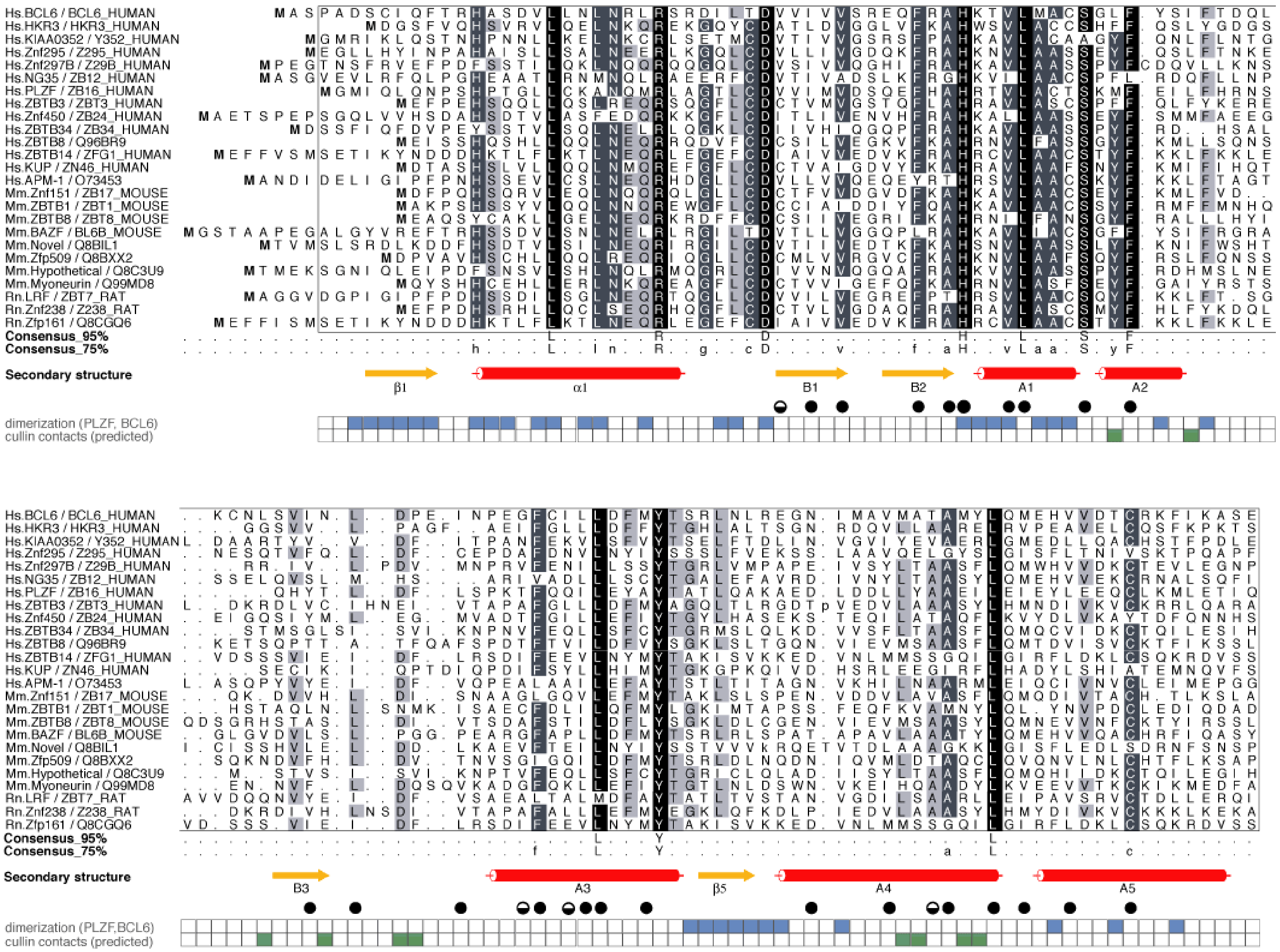

B. BTB domains from BTB-BACK-Kelch (BBK) proteins

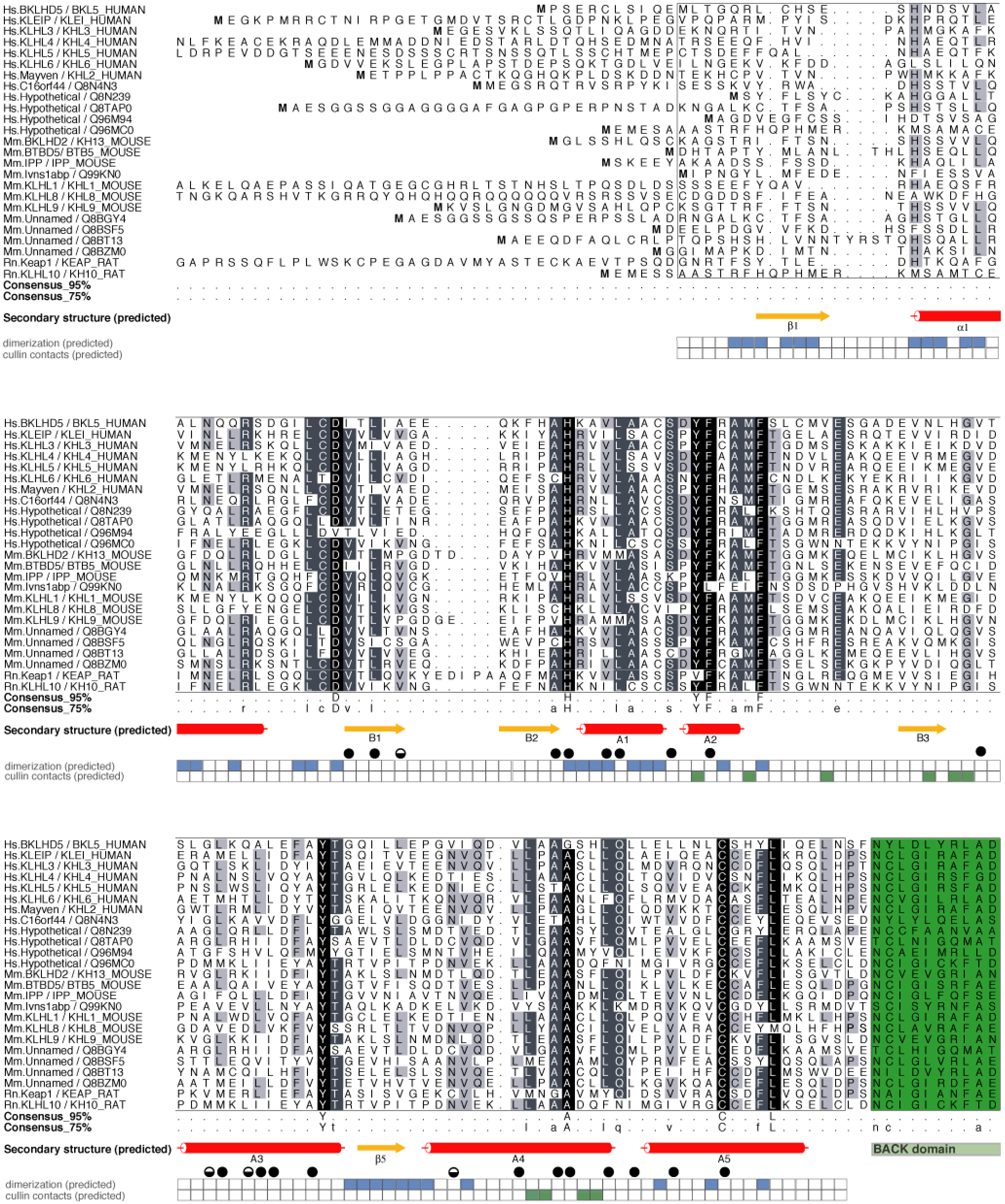

C. Skp1 proteins

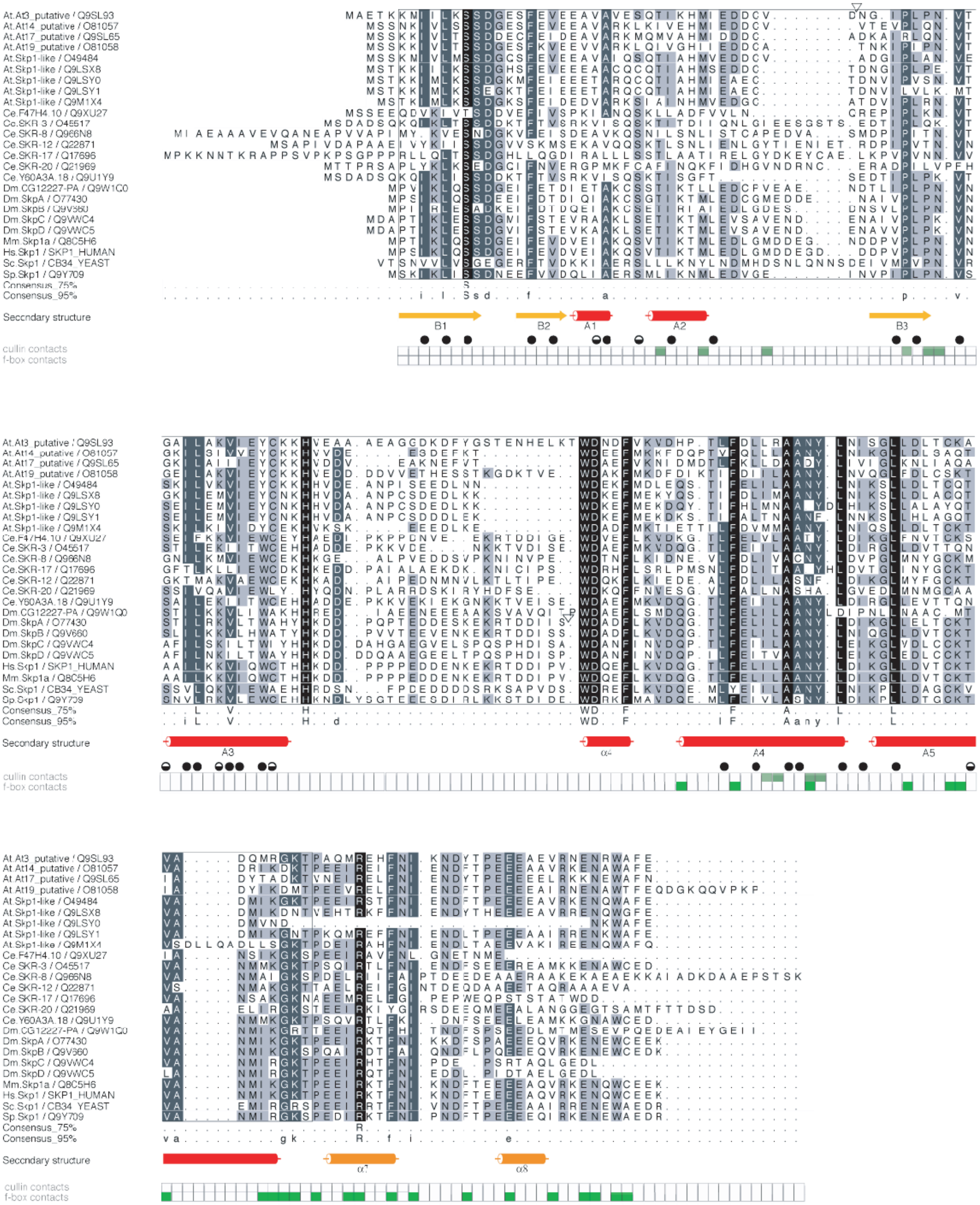

D. T1 domains from T1-Kv proteins

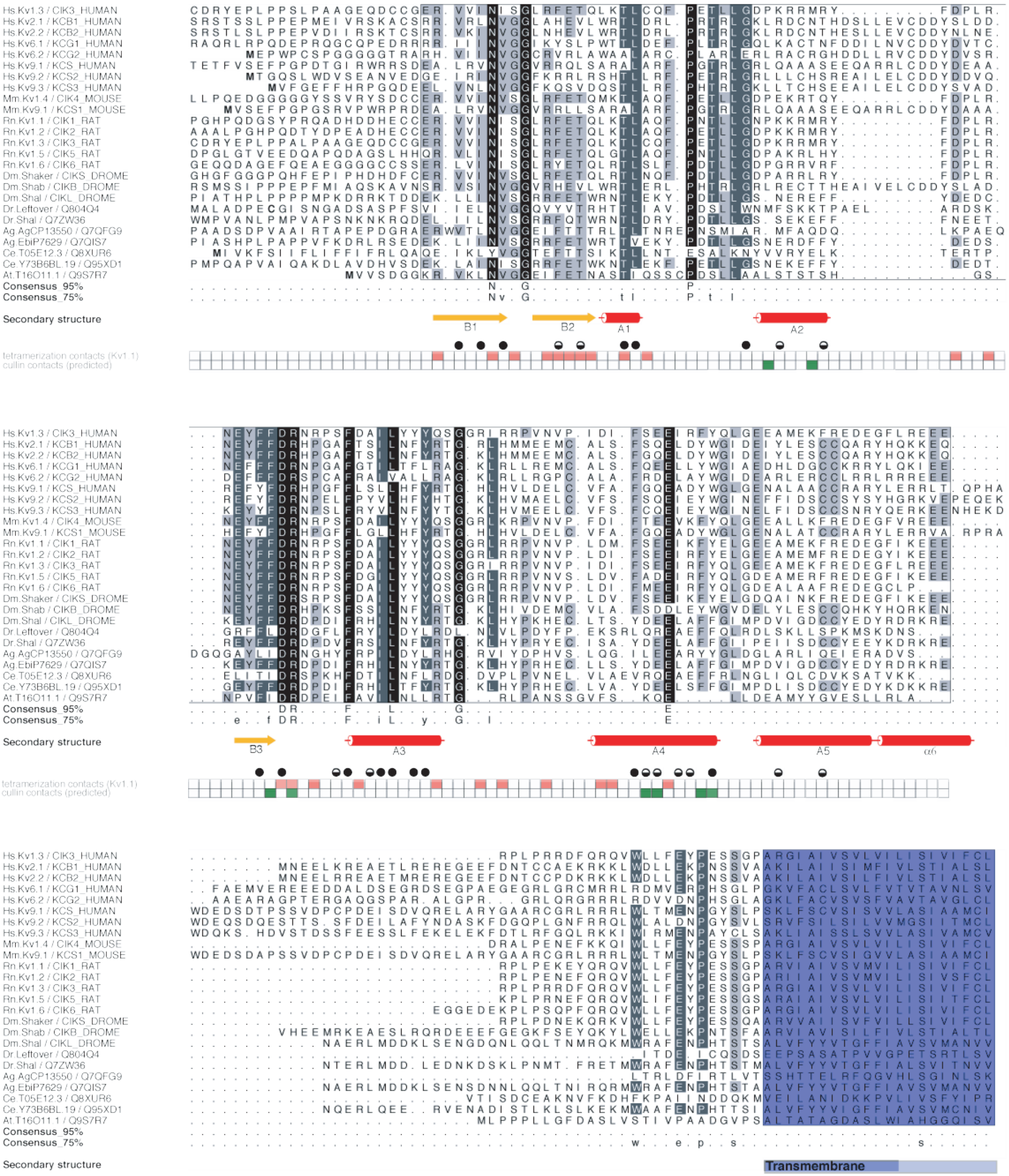

E. BTB domains from MATH-BTB proteins

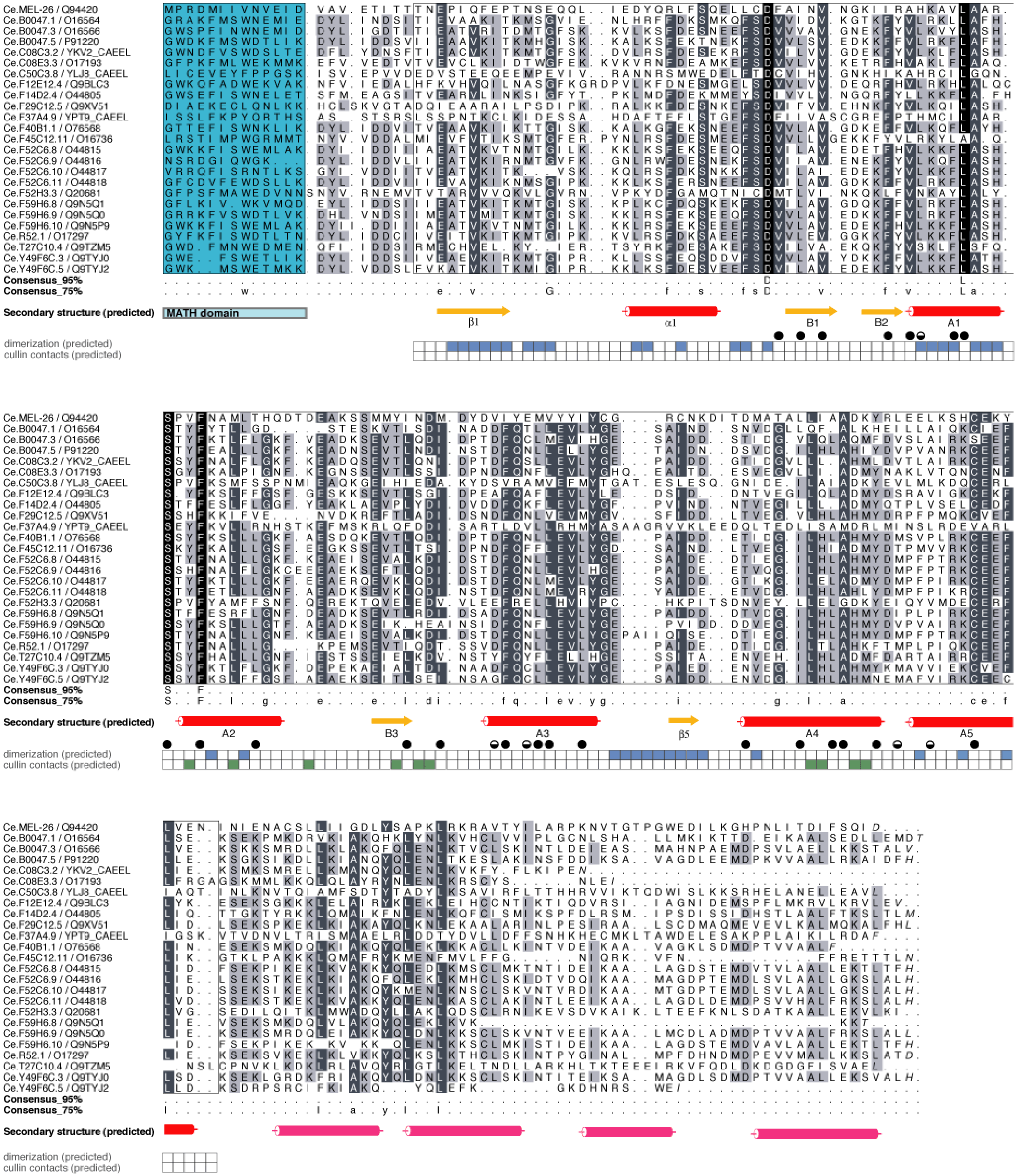

F. BTB domains from BTB-NPH3 proteins

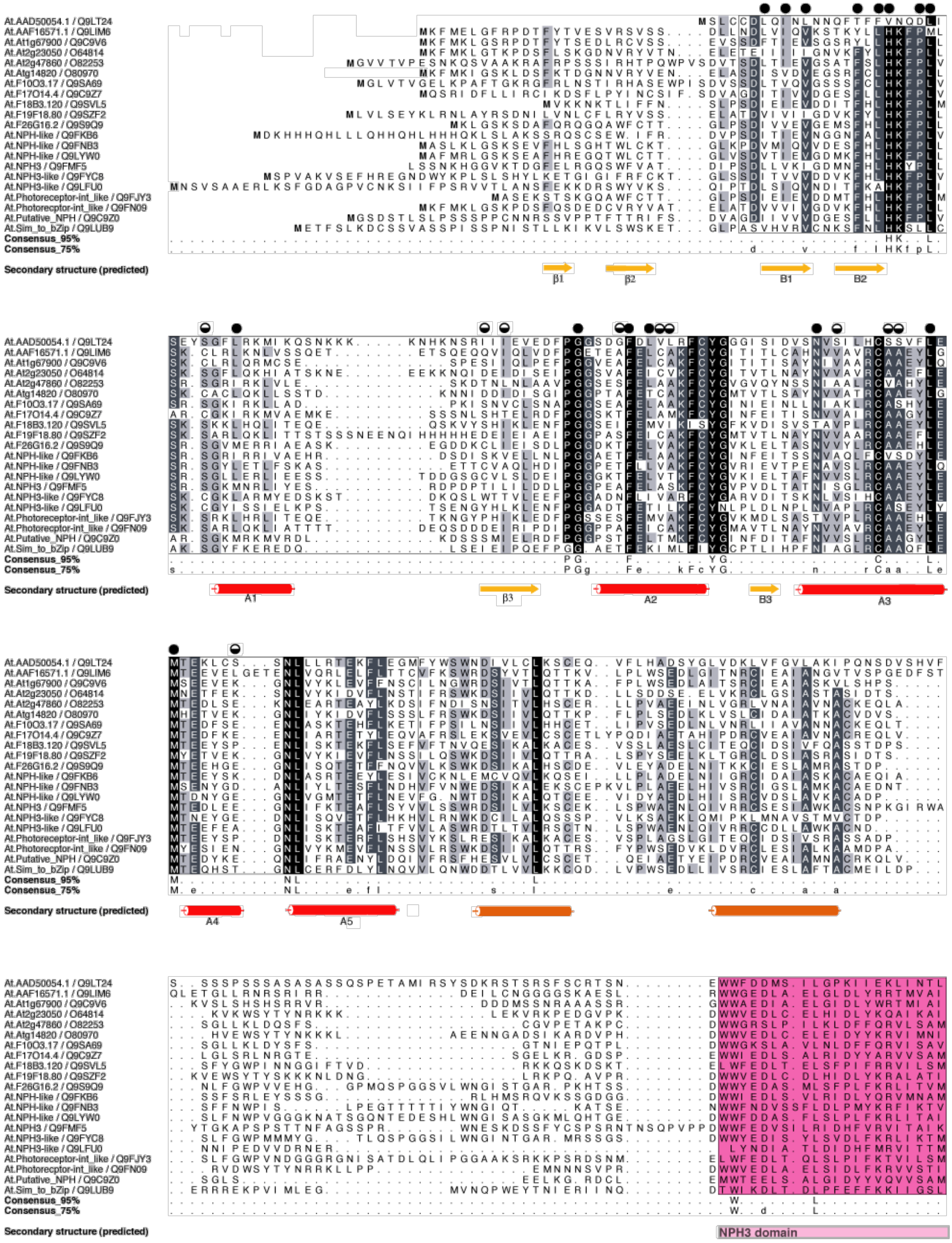

Supplement: Additional data file 1 — Multiple sequence alignment of BTB domains from BTB-ZF, BBK, Skp1, T1-Kv, MATH-BTB and BTB-NPH3 proteins. [file gb-2005-6-10-r82-S1.pdf]
